# Two distinct protein-protein interfaces drive cooperative binding of the herpes simplex virus protein ICP8 to ssDNA, filament formation and annealing essential for viral replication

**DOI:** 10.1016/j.jbc.2025.110498

**Published:** 2025-07-18

**Authors:** Katherine A. Discipio, Jolanta Krucinska, Renata Szczepaniak, Heidi Erlandsen, Andrea M. Makkay, Lee R. Wright, Dennis L. Wright, Sandra K. Weller

**Affiliations:** 1 Department of Molecular Biology and Biophysics, University of Connecticut School of Medicine, Farmington, Connecticut, USA; 2 Department of Pharmaceutical Sciences, School of Pharmacy, Storrs, Connecticut, USA

**Keywords:** herpes simplex virus, ICP8 DNA binding protein, protein-protein interactions, single strand annealing, viral DNA synthesis

## Abstract

Herpes simplex virus (HSV) replicates by forming DNA concatemers using a single strand annealing mechanism mediated by the multifunctional HSV protein ICP8. ICP8 binds cooperatively to form nucleoprotein filaments on ssDNA and promotes annealing of complementary single strands. These functions are believed to be mediated by ICP8:ICP8 protein-protein interactions (PPIs) between the flexible C-terminal domain of ICP8 and the N-terminal domain of a second ICP8 monomer. We previously presented genetic evidence for an ICP8:ICP8 PPI hotspot between two hydrophobic regions and showed that it is essential for the cooperative binding of ICP8 to ssDNA, ICP8 filament formation *in vitro*, and viral replication. This involves interactions between F1142, N1143, and F1144 (FNF) in the C terminal disordered tail of ICP8 and the hydrophobic residues (F843 and W844) in the head region of the N-terminal domain on a second molecule of ICP8. In this article, we propose an additional second distinct PPI hotspot involving residues R922, within the body region of the N-terminal domain of ICP8, and D1087, within the C-terminal domain (CTD). Biophysical studies using thermal melting assays and microscale thermophoresis support an interaction between the ICP8-CTD and the N-terminal domain of ICP8 and show that mutations within each of these hotspots disrupt the interactions. Furthermore, we demonstrate that a construct consisting of the ICP8-CTD can antagonize the assembly of ICP8 filaments *in vitro*, block viral replication compartment formation and virus production, suggesting that these PPI hotspots may be useful in the development of new antiviral therapies.

Herpes simplex virus type 1 (HSV-1) has infected more than 3.7 billion people under the age of 50 years (67% of the world’s population) (1) and is associated with recurrent cold sores, genital lesions, keratitis, corneal blindness, encephalitis, and disseminated neonatal infections. The ability of herpesviruses to establish latent infections that undergo periodic reactivation contributes to their ability to cause life-long disease, viral shedding, and transmission to new hosts. Chronic HSV infections have also been associated with progressive neurodegenerative diseases (2, 3, 4). HSV-1 infections in immunocompromised individuals are far more severe and resistance to current therapeutic agents is on the rise (5, 6, 7) underscoring the need for innovative antiviral strategies.

Herpesviruses replicate their dsDNA by synthesizing concatemers consisting of several viral genomes linked together (8, 9, 10). Concatemer formation is essential because the generation of progeny virus requires packaging machinery that only recognizes longer-than-unit-length molecules of viral DNA. Interestingly, many viruses of bacteria, protozoa, plants, mammals and insects utilize concatemer formation as a replication strategy. All members of this broad and evolutionarily ancient family of viruses harbor a virally encoded two-component recombination system consisting of a processive 5′→3′ exonuclease coupled with a single strand annealing protein (SSAP) (11, 12). The most well-characterized of these systems is λ Redα/β (13, 14), while the analogous two component recombination system in HSV is comprised of ICP8 (SSAP) and UL12, a processive 5′→3′ alkaline exonuclease (15, 16, 17). Redβ can stimulate annealing using short regions of homology, a property that has been exploited for genetic engineering (recombineering) (18, 19). Redβ can also incorporate ssDNA of longer lengths into a growing replication fork (20, 21).

ICP8 (UL29) is a multifunctional metalloprotein that is well conserved in over 130 herpesviruses of mammals, birds, fish, reptiles, amphibians, and mollusks. Human herpesvirus orthologs of ICP8 include UL57 (CMV), ORF 6 (KSHV), and BALF2 (EBV) (22, 23) and was originally thought to function like cellular ssDNA binding proteins such as *Escherichia coli* SSB and eukaryotic replication protein A. These cellular SSBs protect ssDNA from degradation, prevent formation of secondary structures and interact with and stimulate other replication proteins to orchestrate DNA synthesis (24, 25). Indeed, ICP8 was shown to bind ssDNA and stimulate the activities of DNA polymerase, helicase-primase, and the origin binding protein (26, 27, 28, 29, 30, 31). In addition, however, ICP8 belongs to the family of SSAPs (similar to λ phage Red β, and T7 phage gp2.5) in its ability to efficiently promote annealing of complementary ssDNA (32, 33, 34, 35). Combined with recent data, these similarities have led to a model in which HSV uses a SSA mechanism during HSV DNA replication (15, 36, 37, 38, 39). We recently provided additional support for this model by showing that the annealing function of ICP8 is essential for HSV DNA replication (17).

Most, if not all, of the functions of ICP8 are dependent upon the ability of ICP8 to bind in a sequence independent fashion to ssDNA (40, 41). DNA binding is cooperative in nature (ω = 15–38). (40, 41, 42, 43). The cooperative nature of ICP8 binding to ssDNA is well-recognized and considered essential for the formation of a fully coated, extended nucleoprotein filament (“beads on a string”) (40, 41, 42, 43). Both protein:nucleic acid and protein:protein interactions appear to be important for extended filament formation. For example, deletion of the C-terminal tail still permits binding to ssDNA but abrogates cooperativity (41) ICP8 has also been shown to oligomerize *in vitro* in the presence of Mg^2+^ to form double helical protein filaments indicating that protein-protein interactions (PPIs) occur even in the absence of DNA. Furthermore, ICP8 mutants that show a defect in cooperative ssDNA binding are also unable to form double helical protein filaments *in vitro* (17, 44).These observations support the hypothesis that ICP8:ICP8 interactions are essential for cooperative ssDNA binding, annealing, and filament formation.

The structure of HSV-1 ICP8 protein lacking the 60-aa C-terminal tail (ICP8Δ60) has been determined by X-ray crystallography and is comprised of a large N-terminal domain consisting of a head, neck, and shoulder region and a smaller C-terminal helical domain attached through a flexible linker (45). The crystal structure has two molecules of ICP8 in the asymmetric unit, and the C-terminal helical domain of one monomer forms contacts to the back of the head and shoulder regions of the other monomer, interactions which are repeated throughout the entire crystal. Data from our lab and others have suggested that this interaction between the C-terminal domain (CTD) of one monomer and the N-terminal domain of its nearest neighbor is essential for cooperative ssDNA binding and filament formation; however, biophysical evidence and characterization of these interactions is lacking (41, 44, 45). The mapping of residues required for ICP8:ICP8 interactions has been complicated by two factors: (1) the absence of the 60-aa tail in the structure, and (2) the fact that the head, neck, and shoulder regions are interconnected by noncontiguous polypeptide chains such that residues that are distant in the linear sequence can be proximate in the tertiary structure (45). This makes it difficult to express discrete structural domains. In this article, we provide genetic and biophysical evidence showing the presence and characterization of two distinct PPI hotspots that are important for ICP8:ICP8 functions. We also investigated the effects of disrupting these interaction sites on viral replication and on the various biochemical activities of ICP8 including ssDNA binding, filament formation, and ssDNA annealing activity.

## Results

### Identification of two potential PPI hotspots driving ICP8:ICP8 assembly

We previously presented genetic evidence for an ICP8:ICP8 PPI hotspot between residues F1142, N1143, and F1144 in the C terminal disordered tail of ICP8 and hydrophobic residues in the head region on a second molecule of ICP8 (F843 and W844), and we showed that this PPI was essential for viral replication (44). We analyzed two mutations, a triple alanine substitution in F1142, N1143, and F1144 (FNF→AAA) in the tail and a double alanine substitution in F843 and W844 (FW→AA) in the hydrophobic head domain (44). Neither mutant was able to complement an ICP8-null virus for viral growth or replication compartment (RC) formation, indicating that these motifs are essential for ICP8 function. Purified mutant proteins showed functional defects in several biochemical assays. Although both FNF→AAA and FW→AA mutant proteins were capable of binding ssDNA, they were unable to bind cooperatively or to form protein filaments (44). As this interaction was not present in the crystal structure, we utilized an AlphaFold (46) model of the full-length ICP8 protein generated by the Bosse group to better understand the interactions mediated by the 60-aa disordered tail (47).

**Figure 1:**
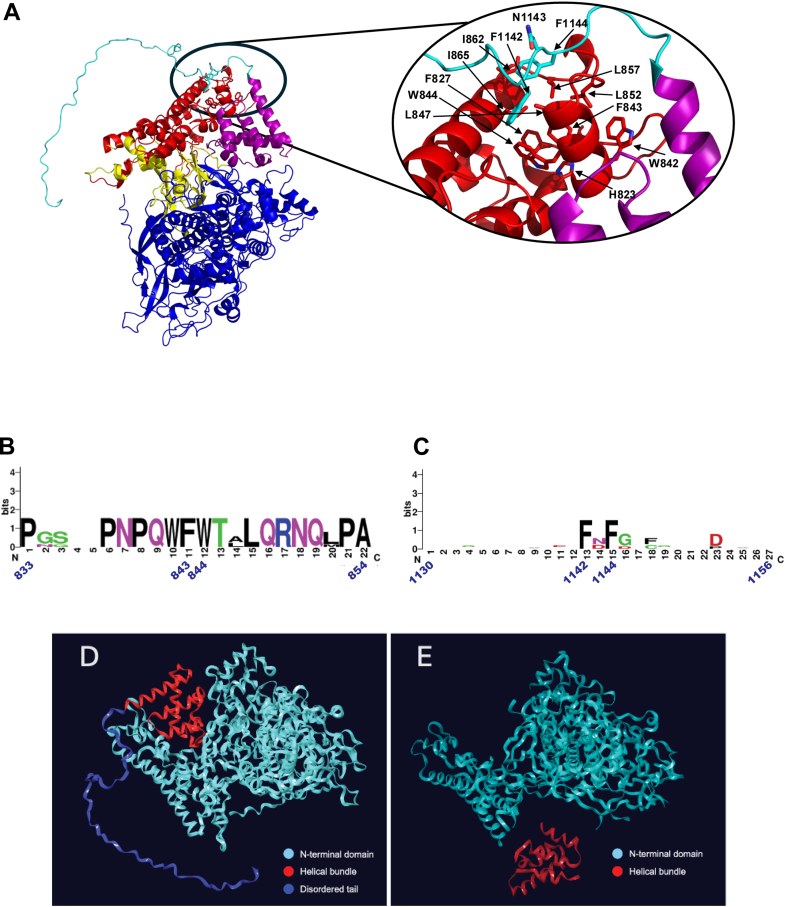
**ICP8 alignments and AlphaFold predictions for interaction between the C terminal disordered region and the hydrophobic head region of ICP8.***A*, (*Left*) structural representation of a full-length monomer of the AlphaFold model of ICP8. The colors used are a simplified version of those used in Mapelli *et al.* in which the “head” region is shown in *red*, the “neck” is shown in *yellow*, the “shoulder” region is shown in *blue*, and the C terminal helical domain is shown in *purple*. The AlphaFold structure of the 60-aa “disordered” tail (residues 1136–1196) is shown in *cyan*. (*Right*) Inset showing the F1141-N1142-F1143 (FNF) motif (*cyan*) in the disordered tail is shown interacting with F843 in the hydrophobic pocket in the head region (*red*). *B* and *C*, conservation of the FNF and FW surrounding regions, respectively between ICP8 homologs of five alpha herpesviruses depicted in WebLogo. The HHV ICP8 homologs are HSV1 (sp|P17470|DNBI), HSV2 (sp|P36384|DNBI), BHV1 (p|P12639|DNBI), SuHV (sp|P11870|DNBI), and VZV sp|P09246|DNBI). Side-by-side comparisons of the AlphaFold (*D*) and crystal structure (*E*) showing the change in relative position between the N-terminal and C-terminal domains. HSV, herpes simplex virus.

The highest ranked AlphaFold model predicts that the C-terminal helical domain (aa 1048–1127) associates with the “head/shoulder” region on the same monomeric unit, unlike the intermolecular interaction observed in the crystal lattice (Fig. 1*A*) (45). The model predicts that the 60-aa tail of ICP8 is largely disordered, demonstrating no discernable secondary structure. The model positions the FNF motif (1142–1144) in contact with a hydrophobic surface comprised of leucine and isoleucine residues (L847, L852, L857, I865, and I867). Beneath this surface is an aromatic amino acid-rich area (H823, F827, W842, F843, and W844) which contains the essential FW pair. This suggests that the FNF and FW motifs interact with one another through the intermediacy of the leucine/isoleucines in a sandwich-like composition. In fact, comparison of sequences for ICP8 homologs from five different alpha herpesviruses showed that all possess a similar motif with either a polar asparagine or aspartic acid residue flanked by the two phenylalanines (Fig. 1*B*). Likewise, there is extensive conservation in the hydrophobic pocket, with 14 completely conserved residues in the head region (Fig. 1*C*). Side-by-side comparison (Fig. 1, *D* and *E*) of the model predicted by AlphaFold and the Mapelli crystal structure (45)underscores the substantial flexibility in the tether region (1030–1047) that controls the relative orientation of the C-terminal and N-terminal domains. This flexibility allows almost identical interactions to form between the FNF motif of the 60-aa tail and a leucine-isoleucine rich patch in the “head region”; either in an intermolecular fashion within the same monomer or in an intramolecular fashion with the adjacent monomer. It is possible that ssDNA or magnesium ions favor the intermolecular interaction and promote the formation of filaments.

**Figure 2:**
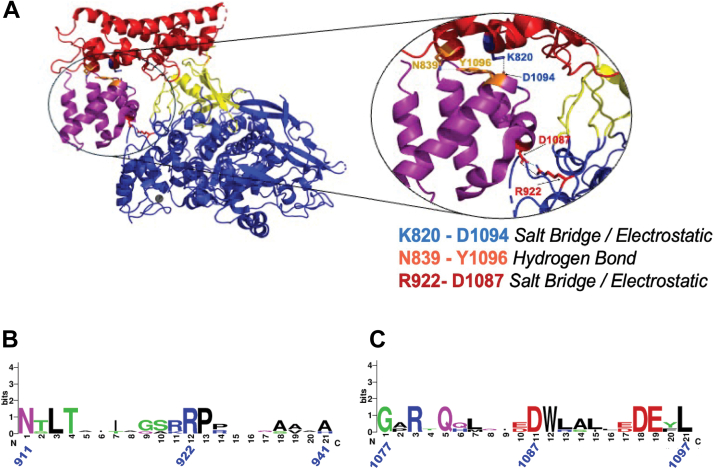
**ICP8 alignments and structure showing potential interactions between the C-terminal helical domain and the head/shoulder region of ICP8.***A*, (*Left*) structural representation of a rotated view of the crystal structure of ICP8 (PDB 1URJ) (42), showing predicted interactions between the C-terminal helical domain (shown in *purple*) and the head (shown in *red*) and shoulder (shown in *blue*). (*Right*) Inset showing interactions between the CTD and N terminus; Lys820-Asp1094 (side chains shown as *blue sticks*); Asn839-Tyr1096 (side chains shown as *orange sticks*) and Arg922-Asp1087 (side chains shown as *red sticks*). *B* and *C*, alignment of the regions surrounding R922 and D1087, respectively, between ICP8 homologs of five alpha herpesviruses as described in the Figure 1 legend. CTD, C-terminal domain; PDB, Protein Data Bank.

**Figure 3:**
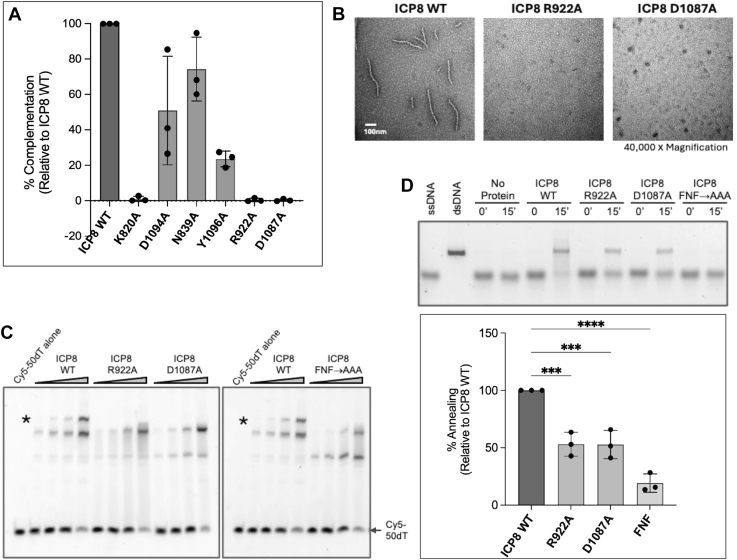
**Analysis of ICP8 mutants.***A*, complementation analysis. Vero cells were transfected with WT or mutant versions of ICP8 and superinfected with HD-2 at an MOI of three for 24 h. Virus was harvested and titered on the ICP8-complementing cell line (S2). Viral titers were normalized to ICP8 WT (100% complementation). Each data point represents an average of normalized viral yields from three independent experiments. Error bars indicate SD of the mean. *B*, the ICP8R922A and ICP8D1087A mutant proteins are unable to form protein filaments *in vitro*. Samples were negatively stained with 2% uranyl acetate and were visualized by electron microscopy at an accelerating voltage of 80 kV. Image represents 40,000 × magnification and size bar is 100 nm. *C*, ability of WT and mutant versions of ICP8 to bind ssDNA. WT and mutant proteins, at increasing concentrations (0, 25, 50, 100, and 200 nM), were incubated with a Cy5-labeled 50-nt ssDNA substrate at 50 nM for 30 min, and the complexes were separated on 5% nondenaturing polyacrylamide gels. The signal was detected using the Bio-Rad ChemiDoc MP imaging system. The *asterix* (∗) represents a supershifted band corresponding to cooperative binding. *D*, ability of ICP8 mutant proteins to carry out annealing of complementary ssDNA as compared to WT protein. Reactions were prepared in DNA annealing buffer containing WT or mutant protein at concentration of 200 nM. Annealing reaction was initiated by addition of 4 Kb ssDNA derived from linearized heat-denatured plasmid at 1 nM concentration followed by incubation at 37 °C. Samples acquired at 0 and 15 min were immediately quenched with termination buffer and analyzed by agarose gel (*top panel*) or fluorescence assay using Pico488 (*bottom panel*). The difference in relative fluorescence units (RFU) between 15 and 0 time point was used for quantification of annealing efficiency normalized to RFU of the reaction with WT protein and presented as a percentage. Each data point represents an average of normalized annealing efficiency from three independent experiments done in duplicates. Error bars indicate SD of the mean. Statistical analyses were performed by one-way ANOVA with the Dunnett’s multiple comparisons test (∗∗∗ indicates *p* < 0.001 and ∗∗∗∗*p* < 0.0001). MOI, multiplicity of infection.

In this article, we also explore a second putative ICP8:ICP8 interaction between the C-terminal helical domain (Fig. 2*A*) of one monomer and a region of the N terminus that spans the head, neck, and shoulder regions of an adjacent monomer, as observed in the crystal structure. Interestingly, identical intramolecular interactions are predicted in the AlphaFold model. Three pairs of interactions (K820:D1094, N839:Y1096, and R922:D1087) are observed between two monomers of ICP8Δ60 in the asymmetric unit of the crystal structure (45). The oval inset in Figure 2*A* shows that residues K820 and N839 in the head region (shown in red) are predicted to contact D1094 and Y1096, respectively, in the C-terminal helical domain (shown in purple). Residue R922 in the ICP8 shoulder (blue), on the other hand, is predicted to contact residue D1087 on the other side of the helical domain (39). To test whether these putative interaction pairs contribute to stabilization of the protein-protein interface, alanine substitution mutations were generated (D1087A, D1094A, Y1096A, R922A, K820A, and N839). Mutant proteins were expressed in Vero cells by transfection, and expression and nuclear localization were confirmed by western blot and immunofluorescence, respectively. All six mutants expressed at WT levels and localized to the nucleus (data not shown), suggesting that they are not globally misfolded. Figure 3*A* shows that variants K820A, R922A, and D1087A failed to complement an ICP8-null virus for viral growth. N839A and D1094A mutations were well-tolerated, while Y1096 showed intermediate levels of complementation. It is possible that the highly conserved E1095 residue that is adjacent residue to D1094 may compensate by forming a salt bridge with K820A, explaining the apparent tolerance of the D1094A mutant (Fig. 2, *B* and *C*). As R922 and D1087 form a discrete interaction pair as predicted by the structure, and both mutants failed to complement the null virus, further investigation focused on these positions. Although sequence alignments of the regions surrounding R922 and D1087 in five HHV ICP8 homologs indicate variability in the adjacent residues, these charged residues appear to be highly conserved (Fig. 2, *B* and *C*).

### Effects of mutations on the *in vitro* behavior of ICP8

We hypothesized that two distinct PPIs are responsible for stabilizing ICP8:ICP8 interactions, one between the C-terminal disordered tail and the head region and another between R922 and D1087 in the C terminus (Fig. 2). To test this hypothesis, mutant ICP8 proteins (R922A and D1087A) were expressed and purified from insect cells infected with recombinant baculovirus and tested for biochemical function. Some assays using the previously reported FNF→AAA mutation (44) are included for comparison.

### Filament formation

ICP8 has been shown to oligomerize to form double helical filaments *in vitro* when incubated in the presence of Mg^2+^ (27, 48). Although the physiological relevance of these double protein filaments remains unknown, the ability of ICP8 to form double helical filaments is routinely used as a proxy for studying ICP8:ICP8 interactions in ICP8-ssDNA nucleoprotein filaments. Both types of filaments are likely to require the ability of one monomer of ICP8 to dock into a neighboring molecule. Negative stain transmission electron microscopy (Fig. 3*B*) shows that WT ICP8 can form double protein filaments while the R922A and D1087A mutants fail to do so, suggesting that these residues are important for filament formation.

### WT and mutant protein binding to ssDNA

We and others have shown that ICP8 mutations that affect filament formation also affect the ability of ICP8 to bind cooperatively to ssDNA (41, 44, 49). An electrophoretic mobility shift assay (EMSA) was used to monitor the ability of ICP8 WT, ICP8 R922A, and ICP8 D1087A proteins to bind cooperatively to ssDNA (50-mer dT oligomer) (Fig. 3*C*). This length oligonucleotide would be predicted to bind four ICP8 molecules ((27). For sake of comparison, the previously reported mutant FNF→AAA was retested (Fig. 3*C*). WT and mutant proteins were incubated at increasing concentrations of protein (0, 25, 100, and 200 nM) with 50 nM of a Cy5-50-mer dT oligo. Figure 3*C* shows that WT and mutant proteins were able to bind ssDNA. We also showed that these variants were able to bind a shorter 14-mer oligo as well as WT, confirming that ssDNA binding *per se* was not affected (data not shown). Cooperative binding can be monitored by EMSA (50) in which the appearance of a supershifted band occurs when there is cooperative blinding. Significantly, while the ICP8 WT protein forms a super-shifted complex (designated with an ∗ in Fig. 3*C*), this band is absent in the mutant samples, suggesting that the mutants are incapable of cooperative binding.

### Annealing

The ssDNA annealing activities of ICP8 WT, ICP8 R922A, ICP8 D1087A, and the previously described ICP8 FNF→AAA mutant were assessed at a protein concentration of 200 nM. The efficiency of annealing of complementary strands of ssDNA was visualized by AGE and measured by PicoGreen fluorescence, as described previously (17, 44). Figure 3*D* shows the ICP8 FNF→AAA mutant exhibited an ∼ 5-fold defect in its ability to anneal complementary ssDNA as compared to ICP8 WT. ICP8 R922A and ICP8 D1087A manifested less severe defects (∼2-fold) in the annealing reaction, suggesting that FNF:FW mediates a more critical interaction.

### Biophysical characterization of PPIs

**Figure 4:**
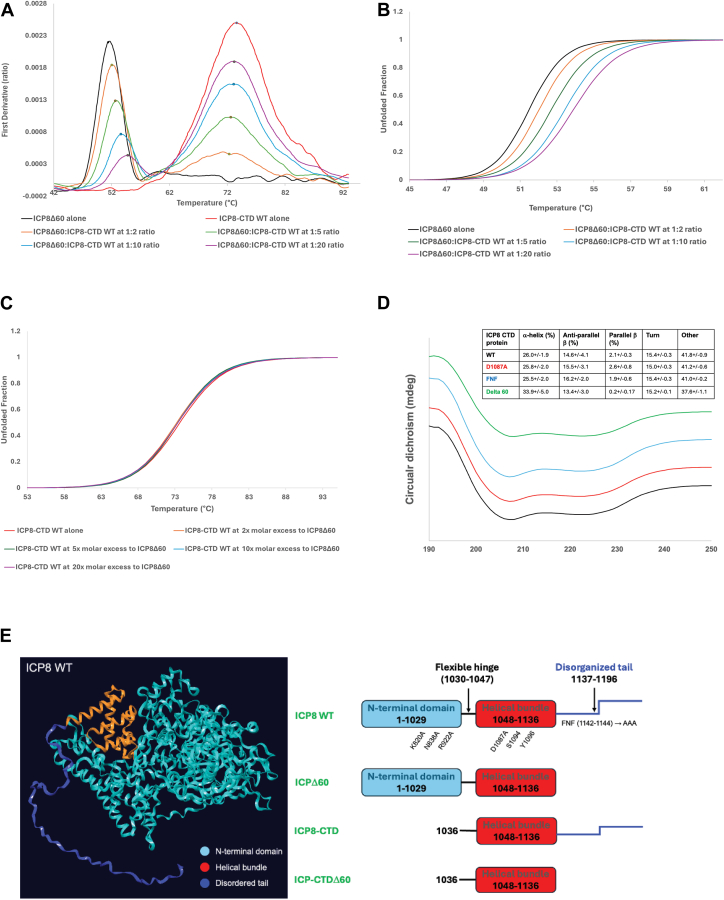
**NanoDSF and MoltenProt binding studies between ICP8Δ60 and an increasing concentration of ICP8-CTD WT.***A*, the first derivative of the fluorescence ratio (ΔF350 nm/ΔF330 nm) as a function of temperature represents an unfolding transition of 5 μM ICP8Δ60 preincubated with 10 to 100 μM of ICP8-CTD WT for 3 h at room temperature. The maximum of the peak corresponds to the inflection point of the underlying ratio curve (inflection temperature, T_i_). The same preparation of proteins was used to perform two independent experiments, done in duplicate and one representative plot for the binding event is displayed. Inset shows a diagram of the ICP8-CTD fragment in which the helical domain is shown in *purple*, and the 60-aa disordered region is shown in *cyan*. *B*, the unfolded fraction of ICP8Δ60 *versus* temperature, derived from the fluorescence-based melting curves in (*A*) and estimated using the two-state reversible unfolding model implemented in MoltenProt. *C*, the concomitant unfolding fraction of a molar excess of ICP8-CTD WT as a function of temperature. *D*, comparative circular dichroism spectra for ICP8-CTD WT and mutant constructs. *E*, comparison of different constructs used in this work. CTD, C-terminal domain.

The ICP8 FNF→AAA and ICP8 FW→AA mutations lie within the 60-aa disordered tail and the hydrophobic pocket on the head region of ICP8, respectively. Based on the phenotypes of these mutants, we theorized that the tail of ICP8 may dock into the FW hydrophobic pocket of an adjacent ICP8 monomer. In this article, we have demonstrated that the new mutants ICP8 R922A and ICP8 D1087A are incapable of complementing the growth of an ICP8 null mutant, forming filaments in solution, binding cooperatively to ssDNA and catalyzing annealing efficiently (Fig. 3). Based on the predicted proximity of residues ICP8 R922 in the head/shoulder region and D1087 in the C-terminal helical domain, we hypothesize that this interaction represents a second ICP8:ICP8 PPI hotspot, one that is required to promote ICP8 oligomerization. Directly studying these interactions in full-length ICP8 WT is complicated by the formation of protein filaments and aggregates. To circumvent these difficulties, we designed a plasmid that would express a His-tagged version of the entire C-terminal domain (ICP8-CTD) (residues 1036–1196) containing the helical bundle as well as the intact 60-aa disordered tail. Codon optimized versions of ICP8 WT and mutant versions (D1087A, FNF→AAA and Δ60) of the ICP8-CTD were cloned and expressed in bacteria as described in Experimental procedures. Figure 4*A* shows a diagram of the ICP8-CTD. We characterized the interaction of the ICP8-CTD WT and mutant constructs with ICP8Δ60 using multiple biophysical techniques.

### Label-free differential scanning fluorimetry

The interactions between the ICP8-CTD fragments and ICP8Δ60 were evaluated using label-free thermal scanning fluorescence (nanoDSF, Tycho N.T.6, NanoTemper Technologies). First, we assessed the thermal stability of the constructs in the absence of binding partner by determining the apparent midpoint transition temperatures, referred to as the inflection temperature (T_i_), which is considered a marker of protein stability (Table 1). The T_i_ for ICP8Δ60 alone was determined to be 50.5 ± 0.2 °C, while the ICP8-CTDs alone were more resistant to thermal melting, yielding T_i_ values in a range of 63 to 75 °C (Table 1 and Fig. S1*A*). Interestingly, a marked difference in the fluorescence spectrum was observed for the ICP8-CTD D1087A mutant relative to the other constructs, with the apparent unfolding transition reduced by ∼ 12 °C. This dramatic shift shows a major thermal effect of substituting aspartic acid with alanine at this position as the thermostability generally depends on subtle changes and the nature of the amino acid residues. Our comparative circular dichroism measurements (CD) of the four ICP8-CTD constructs in the near and far-UV regions revealed no changes in the secondary structure of the CTD D1087A mutant that would account for this difference. The respective ICP8-CTD WT, D1087A, and FNF-AAA fragments are virtually identical in secondary structure composition, displaying the same CD spectra (Fig. 4*D*). Deletion of the last 60 residues in the ICP8 CTDΔ60 construct reflects some degree of gain of secondary structure, with the disordered regions decreased by ∼4%, and the helical content increased by 8%. Furthermore, a distinct thermal denaturation profile was observed for each construct indicating that all four C-terminal fragments are properly folded and when the temperature was decreased after thermal denaturation, all four systems refolded to their initial states. Taken together, a lack of alterations in the overall tertiary fold of the ICP8-CTD D1087A variant but a substantial decrease in its thermal stability relative to the other ICP8 CTD fragments, indicates the importance of the aspartic acid at this position in governing the stability of the native CTD conformations.

**Table 1:** Thermal melting values of ICP8Δ60 and four ICP8-CTD fragments

Sample ID	T_i_ (°C) of the apo form	T_i_ (°C) of the binary ICP8Δ60:CTD complex (at 1:10 M ratio) ± ssDNA		ΔT_i_ (°C) (T_i_ complex-T_i_ apo)	
		- ssDNA	+ ssDNA	-ssDNA	+ ssDNA
ICP8Δ60	50.5 ± 0.2	50.5 ± 0.2	51.6 ± 0.4	n/a	1.1
ICP8-CTD WT	74.0 ± 1.1	52.6 ± 0.1	53.4 ± 0.1	2.1	2.9
ICP8-CTD D1087A	62.8 ± 0.6	51.5 ± 0.1	52.9 ± 0.1	1.0	2.4
ICP8-CTD FNF→AAA	74.8 ± 0.9	51.1 ± 0.2	52.4 ± 0.2	0.6	1.9
ICP8-CTD Δ60	75.0 ± 0.9	51.2 ± 0.2	52.5 ± 0.1	0.7	2.0

*T*_i_ values were generated using the Tycho instruments software from at least three independent experiments.

To assess the interaction of ICP8-CTD constructs with ICP8Δ60, the thermal stability of ICP8Δ60 was determined following incubation with molar excess of the respective ICP8-CTD constructs. All four combinations displayed altered spectrums reflective of the relative strength of the respective binding interactions. (Table 1 and Fig. S2, *A*–*D*). The WT ICP8-CTD had the most pronounced effect on ICP8Δ60 stability during heat treatment with a ΔT_i_ of greater than +2.0 °C. In contrast, the extent of the thermal shift caused by the mutant constructs were less pronounced (CTD D1087A ΔT_i_ = ∼1.0 °C; CTD FNF→AAA ΔT_i_ = 0.6, CTDΔ60 ΔT_i_ = 0.7), suggestive of weaker binding. Finally, the addition of a 19-mer ssDNA oligonucleotide to the preformed binary complexes resulted only in additive stabilization across all four systems (ΔT_i_ of > 1.0 °C), indicating that these specific PPIs in ICP8 are independent of nucleic acid binding to the N-terminal domain.

Next, we investigated the dose dependency of the thermal stability imparted by the ICP8-CTD WT fragment on ICP8Δ60 protein. A fixed concentration of ICP8Δ60 (5 μM) was treated with increasing concentrations of the CTD WT fragment to reach a 1:20 M ratio of ligand to protein. The changes in intrinsic fluorescence emission upon temperature increase were measured for each complex resulting unfolding profiles. These data were subsequently reanalyzed using the MoltenProt web application (https://spc.embl-hamburg.de/) (51) to extract thermodynamic properties, such as heat capacity (ΔCp), alongside the evaluation of T_i_ changes. As anticipated, increases in the concentration of WT CTD were associated with increases in the midpoint transition temperature of ICP8Δ60 and a later onset of unfolding (Table 2 and Fig. 4, *A* and *B*), demonstrating the stabilizing nature of the interaction. No significant change in the T_i_ value was observed for the WT CTD fragment itself (Table 2, Figs. 4*C* and S3*A*), indicating that the CTD does not self-assemble. We repeated this experiment with the other three mutant CTD constructs. Under identical experimental conditions, the ICP8-CTD D1087A fragment had a modest stabilizing effect on ICP8Δ60 (Fig. S3*B*), while no changes in the unfolding profile of the ICP8Δ60 protein were observed with the FNF→AAA CTD and CTD Δ60 mutants (Fig. S3, *C* and *D*), respectively.

**Table 2:** Dose-dependent thermal stability profile of ICP8Δ60 upon binding to an increasing concentration of ICP8-CTD WT fragment

Molar ratio of ICP8Δ60:ICP8-CTD WT	ICP8Δ60:ICP8-CTD WT interactions			
	aT_i_ (°C) ICP8Δ60	bΔCp Component (K) ICP8Δ60	aT_i_ (°C) ICP8-CTD WT	bΔCp Component (K) ICP8-CTD WT
ICP8Δ60 alone	51.5 ± 0.1	−1.11 ± 0.01	n/a	n/a
ICP8-CTD WT alone	n/a	n/a	73.2 ± 0.2	−3.54 ± 0.0
at 1:2 ratio	52.1 ± 0.1	−1.16 ± 0.01	72.8 ± 0.4	−3.46 ± 0.04
at 1:5 ratio	52.7 ± 0.1	−1.21 ± 0.01	73.1 ± 0.1	−3.51 ± 0.01
at 1:10 ratio	53.3 ± 0.1	−1.26 ± 0.01	73.0 ± 0.1	−3.47 ± 0.01
at 1:20 ratio	53.9 ± 0.1	−1.32 ± 0.01	72.9 ± 0.2	−3.50 ± 0.01

a T_i_.

b ΔCp Components were generated by MoltenProt software (https://spc.embl-hamburg.de/).

### Determination of ICP8 binding to the CTD fragment by microscale thermophoresis

**Figure 5:**
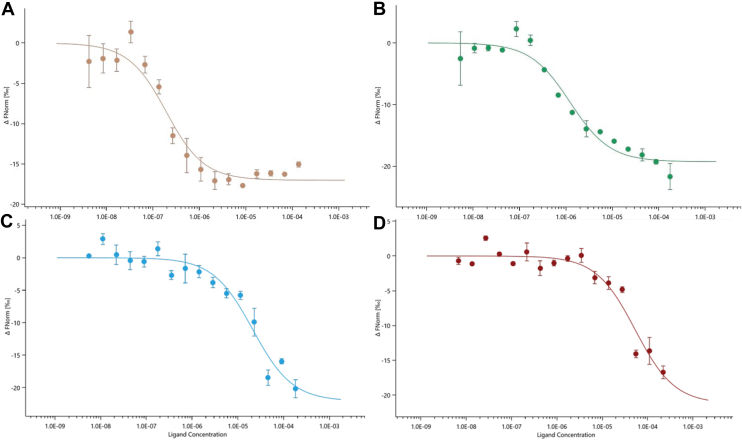
**MST analysis of the interactions between ICP8Δ60 and the ICP8-CTD fragments.** Dose-response curves calculated from the gradual difference of thermophoresis between the fluorescently labeled ICP8Δ60 of both unbound and bound states and plotted as ΔFnorm against the concentration of the unlabeled C-terminal domain constructs. *A*, ICP8-CTD WT fragment. *B*, ICP8-CTD D1087A. *C*, ICP8-CTD FNF-AAA. *D*, ICP8-CTD Δ60 mutant. A single-site binding model accounting for ligand depletion was used to determine dissociation constants, *K*_D_ ± *K*_D_ confidence (±68% confidence interval) using MO Affinity Analysis v3.0.5 software (NanoTemper Technologies GmbH). Error bars represent standard deviation of the average of two values for each concentration and the binding model fit is represented as *solid line*. CTD, C-terminal domain; MST, microscale thermophoresis.

The equilibrium dissociation constant (K_D_) for each pairwise interaction was determined using microscale thermophoresis (Table 3, Figs. 5, *A*–*D*, and S4, *A*–*D*). The purified ICP8Δ60 protein was labeled with amine reactive protein labeling kit RED-NHS Monolith (NanoTemper Technologies MO-L001) using red fluorescence dye NT-647-NHS. The thermophoretic shift was measured with increasing concentrations of the various unlabeled ICP8-CTD constructs. The WT ICP8-CTD construct bound to ICP8Δ60 with a relatively strong affinity (K_D_ = 147 nM [64–335 nM]) which was reduced to varying degrees by mutation of putative hot-spot residues. The binding affinity of CTD D1087A was approximately 12-fold weaker, suggesting that the electrostatic interaction is a considerable stabilizing interaction. The contribution of the FNF motif and other interactions in the disorder tail were even greater as the CTD FNF→AAA and CTDΔ60 mutants were approximately 170- and 400-fold weaker than that of the WT CTD, respectively, with K_D_ values in the high micromolar range.

**Table 3:** Thermophoretic analysis of ICP8Δ60 binding interactions with ICP8-CTD fragments by MST

K_D_ ICP8Δ60:ICP8-CTD fragment		
Target	Titrant	K_D_ (nM) [CI]^a^
ICP8Δ60	ICP8-CTD WT	147 [64–335]
ICP8Δ60	ICP8-CTD D1087A	1553 [939–2568]
ICP8Δ60	ICP8-CTD FNF→AAA	21,107 [11,012–40458]
ICP8Δ60	CTD Δ60	53,500 [24,946–114740]

a Numbers in brackets represent the 68.3% confidence intervals (CI) calculated by the MO. Affinity Analysis software included with the Monolith instrument.

### Ability of mutant and WT versions of the CTD to trans dominantly inhibit filament formation

Mutations of multifunctional proteins such as ICP8 can result in a *trans*-dominant or dominant negative phenotype in which the presence of the mutant protein antagonizes the function of its WT counterpart. This phenotype results from the ability of the mutant protein to perform some of its essential functions while being deficient in others. We previously showed that the FNF→AAA and FW→AA mutants exert a dominant negative inhibitory effect on viral growth and on the ability of WT ICPF8 to form filaments *in vitro* (44). On the other hand, a double mutant bearing both the FNF and FW substitutions had no effect on WT growth or filament formation. These results suggest that the single mutants are capable of creating nonproductive oligomers that interfere with the ability of WT ICP8 to form filaments, whereas the double mutant lacks the ability to form ICP8:ICP8 interactions and therefore could not be incorporated into a growing WT ICP8 filament.

**Figure 6:**
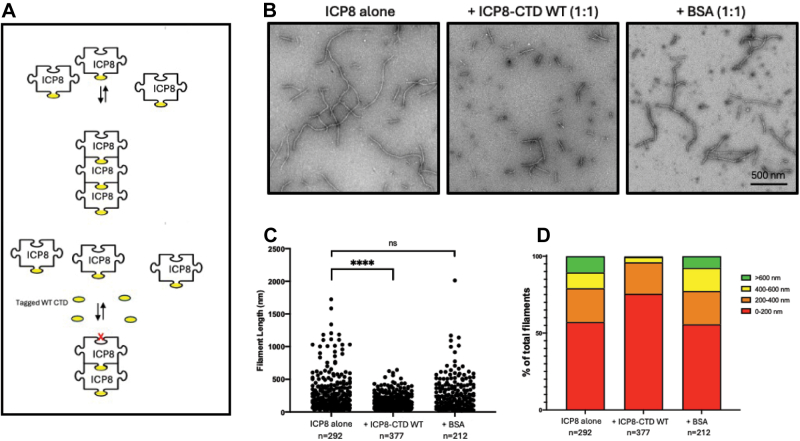
**Filament formation by ICP8 in the presence of ICP8-CTD WT.***A*, model showing filament formation in the presence of ICP8-CTD WT. Filament length is predicted to be shortened in the presence of the inhibitory peptide. *B*, electron microscopy of ICP8 in presence and absence of ICP8-CTD WT. Proteins mixed in 1:1 ratio in a buffer containing 5 mM MgCl_2_ and incubated overnight at 4 °C. Samples were negatively stained with 2% uranyl acetate and visualized by electron microscopy at an accelerating voltage of 80 kV. The scale bars represent 500 nm (magnification, ×60,000). *C*, quantitative analysis of filament length. Filament length was quantified from three randomly chosen 21.4 μm^2^ FOVs (100,00×) using ImageJ software. Statistics showing average filament length were performed by one-way ANOVA (Tukey’s multiple comparison test, ∗∗∗∗*p* < 0.0001, ns = 0.6111). *D*, distribution of filament lengths are shown. CTD, C-terminal domain.

**Figure 7:**
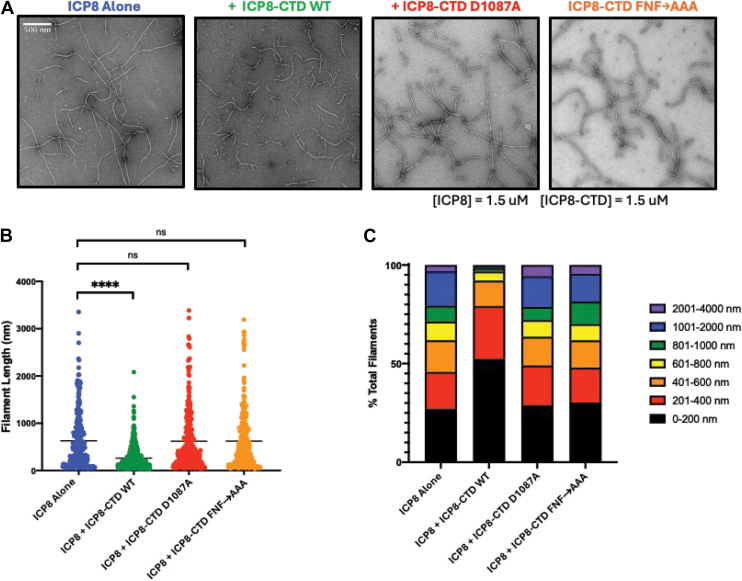
**ICP8 filament length is inhibited by ICP8-CTD WT but not ICP8-CTD D1087A or ICP8-CTD FNF→AAA).***A*, electron microscopy of ICP8 in presence of WT and mutant versions of ICP8-CTD as described in the legend to Figure 6. *B*, quantitative analysis of filament length. Filament length was quantified as described in the legend to Figure 6. Statistics on average length were performed by one-way ANOVA (Tukey’s multiple comparison test, ∗∗∗∗*p* < 0.0001). *C*, distribution of filament lengths is shown. CTD, C-terminal domain.

In this article, we performed a similar experiment using full-length ICP8 in the presence of the WT ICP8-CTD fragment described above. Since this fragment encompasses the helical domain containing D1087 and the C-terminal disordered tail containing FNF, we predicted that it would be able to bind to WT ICP8 at both the head/shoulder and the FW pocket on the head and interfere with filament formation as depicted in Figure 6*A*. Figure 6, *B* and *C* show that when full-length ICP8 was incubated alone or in the presence of bovine serum albumin (BSA), a significant number of filaments in the 200 to 600+ nm length were observed; however, in the presence of WT ICP8-CTD, filament formation was severely abrogated, and the length of the filaments was less than 200 nm in length. Figure 7 shows that WT ICP8-CTD can significantly decrease filament length; however, the presence of mutant versions containing either ICP8-CTD D1087A or ICP8-CTD FNF→AAA resembles WT in the absence of a CTD. This experiment confirms the importance of both D1087 in the C-terminal helical domain and FNF in the C-terminal disordered tail in filament formation.

### Ability of WT and mutant versions of the ICP8-CTD to interfere with viral infection

**Figure 8:**
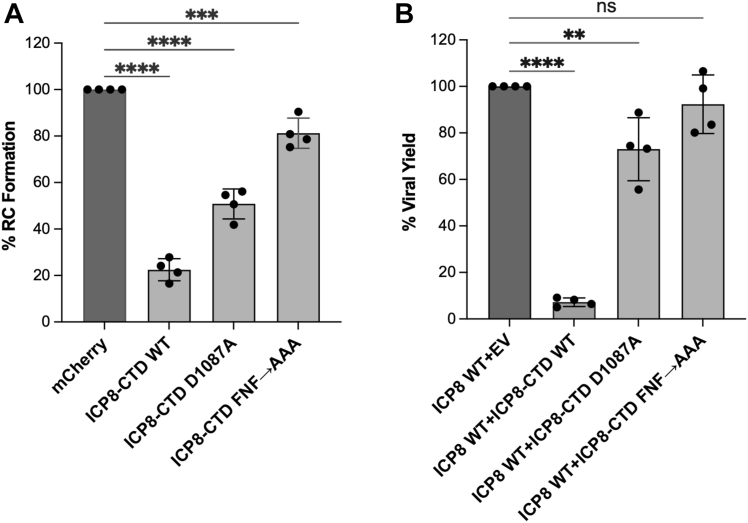
**Efficiency of viral replication in the presence and absence of *trans*-dominant peptides.***A*, replication compartment formation in KOS-infected Vero cells in the presence of transiently expressed WT and mutant versions of ICP8-CTD as assessed by immunofluorescence staining. The ratio of transfected and infected cells containing RC to the total number of cells was normalized to the mCherry control ratio. Each data point represents an average of 50 to 200 transfected and infected cells counted for each transfected plasmid across four independent experiments. Error bars indicate SD of the mean. Statistical analyses were performed by one-way ANOVA with the Dunnett’s multiple comparisons test (∗∗∗∗ -*p* < 0.0001, ∗∗∗ - *p* = 0.0007). *B*, complementation of ICP8 null (HD-2) virus by transiently expressed full-length ICP8 protein in the presence of empty vector (EV, control) or ICP8-CTD (WT, D1087A, or FNF) constructs. Transfected cells were superinfected with HD-2 virus 14 to 16 h post transfection for 24 h. Viral yields from infected cells expressing ICP8-CTD (WT or mutant versions) were normalized to the EV control and presented as percentage. Each data point represents an average of normalized viral yield from four independent experiments, and error bars indicate SD of the mean. Statistical analyses were performed by one-way ANOVA with the Dunnett’s multiple comparisons test (∗∗∗∗ -*p* < 0.0001, ∗∗ - *p* = 0.0039, ns – *p* = 0.5338). CTD, C-terminal domain; RC, replication compartment.

The observation that ICP8-CTD antagonized filament formation (Figs. 6 and 7) led us to ask whether expression of ICP8-CTD in the context of viral infection would be inhibitory. ICP8 is one of the key components in viral genome replication and is required for the formation of RCs (52). To track transfected cells that express the ICP8-CTD constructs, a Flag-tag mammalian expression plasmid for WT and mutant versions of ICP8-CTD was used. Vero cells were first transfected for 16 h, either with Flag-tagged versions of the CTDs or a plasmid expressing mCherry to control for the effects of transfection on infection efficiency. Cells were then infected for 7 h with KOS at a multiplicity of infection (MOI) of 10 plaque forming units per cell (PFU/cell), and infected cells with or without RCs were detected by immunofluorescence staining using antisera specific for the HSV replication protein, UL42. The fraction of RC-positive transfected and infected cells in the mCherry control were normalized to 100% and compared to the fraction of RC-positive transfected and infected cells in which WT or mutant versions of the ICP8 CTD was present. Figure 8*A* shows that RC formation was inhibited up to 98% in cells transfected with ICP8-CTD WT; whereas RC formation was inhibited to a much lesser extent by ICP8 CTD D1087A (49%) or ICP8 CTD FNF→AAA (19%).

Next, we asked whether the presence of ICP8-CTD could inhibit infection using a complementation assay, which measures the ability of transiently expressed ICP8 WT to complement the growth of an ICP8 null virus (HD2). In this experiment, Vero cells were first transfected for 16 h with plasmids expressing ICP8 WT and either WT, D1087A or FNF→AAA mutant versions of Flag-tagged ICP8-CTD. Cells were then superinfected with the ICP8 null virus at an MOI of 5 PFU/cell for 24 h. Virus production was measured on an ICP8-expressing cell line (S2). Coexpression of full length ICP8 with ICP8-CTD WT resulted in 93% inhibition of virus production (Fig. 8*B*). On the other hand, the ICP8-CTD D1087A mutant version of the CTD reduced the virus yield only by 27%, and expression of ICP8 CTD FNF→AAA was similar to that of the empty vector (EV) control.

## Discussion

The existence of two independent PPI interaction hotspots was confirmed using genetic, biochemical, and biophysical approaches. We previously reported genetic evidence for a PPI hotspot between the C-terminal disordered tail (FNF) and the hydrophobic head region (FW) of ICP8, and we now describe a second PPI hotspot between R922 in the helical region of the C terminus and residue D1087 in the shoulder region. Mutations in these residues (R922A and D1087A) were defective in the growth of an ICP8 null virus. Mutant proteins were likewise deficient in filament formation, cooperative DNA binding and annealing of ssDNA. A 160-aa ICP8 C-terminal domain construct (ICP8-CTD) consisting of the helical domain (aa 1049–1129) and the disordered C-terminal tail (1136–1196) was used to probe ICP8:ICP8 interactions. Biophysical experiments demonstrated direct binding between ICP8-CTD and ICP8Δ60, while CTDs bearing either the D1087A or the FNF→AAA tail mutations showed little or no binding, consistent with our genetic data. The data also reveal that the hydrophobic interaction mediated by the FNF motif contributes substantially more stability to the PPI compared to the electrostatic interaction mediated by D1087. In this article, we have shown that several ICP8 residues are necessary for stabilization of ICP8:ICP8 self-association and are required for all major functions of ICP8 including filament formation, cooperative binding to ssDNA, and annealing of complementary ssDNA. Consistent with our observations, elimination of these interactions leads to severe defects in RC formation, HSV DNA replication and virus production.

Several lines of evidence support a model in which the HSV exo/SSAP (UL12 and ICP8) stimulate SSA-mediated DNA replication: (1) UL12 and ICP8 can drive strand transfer reactions over distances of at least 7kb *in vitro* (37, 38); (2) using recombination reporter assays in HSV-infected mammalian cells, SSA was increased, while homologous recombination (HR) and nonhomologous end joining (A-NHEJ) were decreased (15); (3) the increase in SSA was abolished when cells were infected with a viral mutant lacking UL12, and expression of UL12 alone caused an increase in SSA (15). (4) Like λ Red β, ICP8 binds cooperatively to ssDNA forming nucleoprotein filaments that resemble “beads-on-a string” (33, 34, 53). These results have led to a model for HSV DNA replication in which UL12 and ICP8 stimulate SSA to create recombination intermediates that can be resolved into concatemeric DNA prior to packaging into infectious virus (15, 37, 38, 39). This model is supported by our finding that an ICP8 mutant that maintains ssDNA binding but could not promote annealing of complementary ssDNA failed to synthesize viral DNA (17).

In addition to providing greater insight into the important role that these protein:protein interactions play in HSV DNA replication, they also reveal a new pathway that may be targeted for antiviral therapy. The finding that the ICP8-CTD produced a pronounced antiviral effect when expressed in an HSV infected cell provides strong evidence that more drug-like therapeutic agents that disrupt the interface between molecules of ICP8 could also be antiviral. Moreover, the identification of these distinct hot-spot interactions that drive filament formation can enable the application of structure-based drug design or screening approaches to identify small molecule leads for inhibitors. An attractive feature of targeting these hot-spots for drug development is that these interactions are likely to be unique to ICP8 and not present in host SSB or SSAP proteins, providing a built-in level of selectivity that would be difficult to achieve if targeting the protein:nucleic acid interactions. In addition, as these agents would be mechanistically distinct from current HSV inhibitors, they would provide an important option in ACV-resistant variants as well as for use in combination therapy. Efforts to identify small molecule and peptide-based inhibitors of ICP8 filament formation are currently ongoing.

## Experimental procedures

### Cell lines and viruses

The HSV-1 KOS strain was used as WT in all experiments. African green monkey kidney (Vero) cells were purchased from the American Type Culture Collection and maintained as monolayers in Dulbecco's modified Eagle's medium (Invitrogen) supplemented with 5% fetal bovine serum and 0.1% penicillin–streptomycin. The ICP8-null virus, HD-2, containing a LacZ insertion mutation in the ICP8 gene and the ICP8-complementing cell line S2 were generously provided by David Knipe (Harvard University, Boston, MA). S2 cells were maintained under G418 selection (400 μg/ml). All cell lines were maintained under a humidified atmosphere containing 5% CO_2_.

### DNA constructs and mutagenesis

Mammalian expression of ICP8 utilized the pSAK-ICP8 vector (44), containing the full-length ICP8 gene under control of the CMV promoter. Construction and generation of recombinant baculovirus stocks for protein expression in *Sf9* cells were performed using pFastBac1-ICP8 which contains the full-length ICP8 gene (KOS) under control of the polyhedrin promoter, while pFastBac1-ICP8Δ60 contains the ICP8 gene with a 60 amino acid C terminal deletion. For mammalian expression of the C-terminal domain (ICP8-CTD), pcDNA3-Flag-ICP8-CTD was created by cloning the CTD sequence (aa 1049—1135) with a Flag-tag into pcDNA3 at the BamHI and EcoRI restriction sites. To generate pET28a-His-ICP8-CTD for *E. coli* expression, a codon optimized version of ICP8-CTD (GenScript) was cloned into the pET28a vector at the BamHI and EcoRI restriction sites. Alanine substitution mutations (R922A, D1087A, FW→AA and FNF→AAA) were generated in pSAK-ICP8, pcDNA3-Flag-ICP8-CTD, pFastBAC1-ICP8, and pET28a-His-ICP8-CTD using the QuikChange II XL Site-Directed Mutagenesis Kit according to the manufacturer’s suggested protocol (Agilent Technologies). pET28a-His-ICP8-CTDΔ60 vector for expression of CTDΔ60 was generated using In-Fusion cloning (Takara). The accuracy of WT and mutant versions of the ICP8 gene was confirmed by sequencing.

### Antibodies and reagents

Polyclonal rabbit anti-UL42 833 was kindly provided by Deborah Parris (Ohio State University, Columbus), monoclonal mouse anti-Flag M2 and Alexa Fluor secondary antibodies goat anti-mouse 594, goat anti-mouse 488, and goat anti-rabbit 488 used for immunofluorescence were acquired from Molecular Probes. Hoechst 33258 was used as a nuclear marker dye.

### Protein purification

WT and mutant versions of full-length ICP8 as well as ICP8Δ60 proteins were purified from *Sf9* insect cells infected with recombinant baculovirus bearing various ICP8 sequences (37). Infected cell pellets were resuspended in swelling buffer (10 mM Tris–HCl [pH 7.5], 10 mM KCl, and 1.5 mM MgCl_2_) with 1× EDTA-free protease inhibitor cocktail (Roche, catalog no. 05056489001). After 30 min incubation on ice, cells were lysed with a Dounce homogenizer, nuclei were pelleted at 5000*g* and 4 °C for 8 min and extracted with extraction buffer (swelling buffer plus 1.2 M NaCl and 1× protease inhibitor cocktail) on ice for 40 min. Nuclei were harvested by centrifugation at 32,000 rpm (Ti70 fixed-angle rotor, Beckman Coulter ultracentrifuge) for 40 min at 4 °C. Since ICP8 R922A and ICP8 D1087A proteins are present in cytoplasmic and nuclear fractions, whole cell lysates were cleared by centrifugation at 15,000*g* rpm for 40 min at 4 °C. All cleared samples were dialyzed over night at 4 °C (12,000–14,000 molecular weight cutoff membrane) into low salt ICP8 buffer (20 mM HEPES-KOH [pH 7.5], 100 mM NaCl, 10% [v/v], 1 mM DTT, and 0.1 mM EDTA), filtered through polyvinylidene fluoride 45 mm filter and run on HiLoad 16/10 SP-Sepharose cation-exchange FPLC column. Elution from the column was performed using a linear gradient (80 ml elution, flow rate 1 ml/min) to 100% high salt ICP8 buffer (20 mM HEPES-KOH [pH 7.5], 1 M NaCl, 10% [v/v] glycerol, 1 mM DTT, and 0.1 mM EDTA). Fractions were analyzed by Coomassie stained SDS-PAGE gel and optimal fractions were individually dialyzed overnight at 4 °C back into the low salt ICP8 buffer and flash-frozen for long-term storage at −80 °C.

For *E. coli* expression, BL21 cells were transformed with pET28(a)-His-ICP8-CTD vectors expressing WT and mutant ICP8-CTD proteins. Transformed cells were grown in LB (1 L) containing kanamycin at 37 °C until culture reached an *A*_600_ of 0.6 to 0.8, at which point IPTG was added (final concentration of 1 mM). At 18 to 20 h post induction, cells were pelleted, and pellets were reconstituted in 50 ml of lysis buffer [20 mM sodium phosphate buffer pH 7.5, 100 mM NaCl, 5 mM β-mercaptoethanol, 10% [v/v] glycerol, and 1 mM phenylmethylsulfonyl fluoride] and lysed by sonication (45% amplitude, 10 s on, 30 s off, 2 min total sonication time). Cell lysates were cleared by centrifugation at 15,000 rpm in SS-34 rotor for 30 min at 4 °C, filtered through a 0.45 μm filter and added to the column with Talon Cobalt beads (∼15 ml resin volume) equilibrated with ∼5 CV (75 ml) of wash buffer (lysis buffer with 10 mM imidazole). The column was capped and incubated for 30 min shaking at 4 °C. Flow through was collected, and column was washed 2× with 50 ml wash buffer. Protein was eluted with 5 × 10 ml fractions of elution buffer (lysis buffer with 300 mM imidazole). Fractions were analyzed on Coomassie stained SDS-PAGE gel, combined and concentrated to ∼1.5 ml in a 10K Amicon concentrator. Concentrated sample was run on a gel filtration column (Superose 12, 10/300 column, 0.5 ml/min flow rate, 0.5 ml fractions). Peak samples were collected and analyzed on Coomassie-stained SDS-PAGE gel and the peak fractions were flash-frozen for long-term storage at −80 °C in storage buffer (20 mM sodium phosphate pH 7.5, 100 mM NaCl, 2 mM DTT, 0.1 mM EDTA, and 10% [v/v] glycerol). All protein concentrations were assessed by Bradford assays.

### Immunofluorescence analysis

Cells grown on 12 mm glass coverslips were transferred to a 24-well plate and washed with 1× PBS, followed by fixation for 10 min in 4% paraformaldehyde and subsequent washing with 1× PBS. Cells were permeabilized with 1% Triton X-100 for 10 min, washed with 1× PBS and blocked in 3% normal goat serum for 1 h at room temperature (RT). All the solutions were prepared in 1× PBS. Primary and secondary antibodies were diluted in 3% normal goat serum. Coverslips were overlayed with 130 μl of antibody dilution and incubated for 1 to 1.5 h at RT with rocking, followed by extensive washing with PBS. Coverslips were incubated with 130 μl of secondary antibody dilution for 40 min at RT with racking protected from light. Secondary antibody was replaced with 500 μl of 1:10,000 dilution of Hoechst stain for 15 min. After extensive washing with PBS, coverslips were mounted on slides using Fluoroshield mounting medium (Sigma-Aldrich).

### Transient complementation assay

Transient transfection complementation assays were performed as previously described (44). Briefly, Vero cells were grown in 12-well plates to 70 to 80% confluency and transfected with 50 ng of a plasmid expressing ICP8 (WT or mutant) and 500 ng of carrier DNA, pUC119, using Lipofectamine Plus reagent (Invitrogen) according to manufacturer’s protocol. Transfection with EV, pSAK was used as a background control. At 18 h post transfection, cells were infected with HD-2 at an MOI of 3 PFU/cell for 1 h at 37 °C; inoculum was removed, cells were washed with 1× PBS and overlayed with 1 ml of growth medium. At 24 hpi, media and cells were harvested, subjected to three cycles of freezing and thawing and viral yields were determined by titration on ICP8 expressing cells (S2). The percent complementation was calculated according to the formula: (viral yield _mutant ICP8_ − viral yield _EV_)/(viral yield _WT ICP8_ − viral yield _EV_) × 100.

### Filament formation

WT ICP8 and full-length D1087A and R922A mutant proteins (0.5 μM) were incubated overnight (4 °C) in ICP8 storage buffer (20 mM HEPES-KOH [pH 7.5], 100 mM NaCl, 10% [v/v] glycerol, 1 mM DTT, 0.1 mM EDTA, and 5 mM MgCl_2_). Samples were diluted 1:5 in water, absorbed onto Formvar-carbon-coated 300-mesh copper grids and negatively stained with 1%-2% uranyl acetate. Images were acquired using a Hitachi H-2650 transmission electron microscope at accelerating voltage of 80 kV.

### Electrophoretic mobility shift assay

Purified WT or mutant proteins (0–200 nM) were incubated with a (50 nM) Cy5-labeled 50-mer ssDNA oligo (IDT) in 20 mM HEPES (pH 7.5), 150 mM KCl, 2 mM EDTA, 1 mM TCEP [tris(2-carboxyethyl)phosphine], and 6% [w/v] Ficoll and incubated on ice for 30 min. Bound and unbound DNA species were separated on a 5% nondenaturing polyacrylamide gel (29:1 acrylamide:bis-acrylamide) in 1× TBE at 60V and 4 °C and imaged using a ChemiDoc MP Imaging System (Bio-Rad). Gels were briefly fixed in 10% ethanol in 1× TBE prior to imaging.

### Annealing of complementary ssDNA

A linearized heat-denatured dsDNA plasmid (4 Kb) was used to create ssDNA. Plasmid DNA was linearized by digestion with PstI (New England biolabs, Inc) and phenol:PCI purified and EtOH precipitated. Prior to the annealing reaction, linearized dsDNA was diluted in water and denatured at 95 to 98 °C for 3 min. Reactions of 18 μl total volume were prepared in DNA annealing buffer containing 20 mM Tris–HCl pH [7.5], 50 nM NaCl, 5 mM MgCl_2_, 1 mM DTT, and 0.1 mg/ml BSA with 200 nM of either WT or mutant ICP8 protein. Reactions were initiated by addition of ssDNA at a final concentration of 1 nM and incubated at 37 °C. Samples were removed at 0 or 15 min and quenched by the addition of 4× termination buffer (final concentration of 45 mM EDTA [pH = 8] and 0.1% [w/v]. Annealing reaction products were visualized on a 1% agarose gel, stained with SYBR Gold reagent (Invitrogen, catalog no S11494) and imaged using ChemiDoc MP Imaging System (Bio-Rad). Quantification of the dsDNA annealing products was performed using Pico488 fluorescent dye (Lumiprobe) was used according to the manufacture’s specifications in 96-well Fluotrac 200 black plates (Greiner Bio-One). Fluorescence was measured using a SpectraMax 3 plate reader at the following wavelengths: excitation 485 nm, emission 520 nm, and cutoff 530 nm. To calculate the percent annealing of the mutants, relative fluorescence units (RFU) were normalized to that of the WT ICP8 sample, representing 100% annealing. Background relative fluorescence units at the 0 min time point was subtracted for all samples.

### CD of the ICP8 CTD constructs

The four purified ICP8-CTD constructs (WT, D1087A, CTD FNF, and Δ60) were buffer exchanged into 20 mM sodium phosphate pH 7.6, followed by dilution to 0.2 to 0.3 mg/ml to ensure good quality of the signal. All protein concentrations were determined by tryptophan, tyrosine, and cystine absorbance. The same molar extinction coefficient of the ICP8-CTD WT enzyme was used for the two mutants, D1087A and FNF as the amino acid substitutions do not alter the content of chromophore groups. The CD spectra was recorded with a Chirascan V100 CD spectrometer in the range of 190 to 280 nm at RT, with an incident bandwidth of 1 nm, a path length of 0.1 cm, and a scan rate of 3 sek (time per point). Measurements were performed in triplicates, and the data were analyzed using Beta Structure Selection server (BeStSel) available at https://bestsel.elte.hu. The average values and standard deviation are presented in Figure 4*D*. Representative spectra for each construct were purposedly offset to avoid overlapping.

### Label-free thermal shift assays

Label-free thermal shift assay experiments of ICP8 were performed with a Tycho NT.6 instrument (NanoTemper Technologies). The samples at 5 μM concentration were heated in a glass capillary at a rate of 30 °C/min, and the intrinsic tryptophan/tyrosine fluorescence at 330 and 350 nm was recorded. Data analysis, data smoothing, and calculation of derivatives were conducted using the internal evaluation features of the Tycho instrument. Thermal shift assays were performed in 20 mM Na Phosphate [pH 7.6], 100 mM NaCl, 100 μM EDTA, 1 mM Tris(2-carboxyethyl)phosphine (TCEP), 10% [v/v] glycerol, and 5 mM MgCl_2_. The effect of addition of the *C*TD fragments to ICP8Δ60 was also probed in the absence or presence of 1.2 M excess of ssDNA (5′–GCT GTT GAG GTT GCG CAT G–3′).

### Thermal stability estimation by MoltenProt

The raw fluorescence data generated by the NanoTemper instrument were uploaded and preprocessed by FoldAffinity by selecting temperature windows for concomitant unfolding events, fixing it to the 45 to 61 °C range for the first transitions and to the 53 to 93 °C range for the second transitions and applying smoothening transformations (54). Next, the melting curves were fitted using a reversible two-state model to yield the enthalpy of unfolding ΔHu, ΔC_p_ and the melting temperature T_i_. These parameters were then used to estimate the fraction of unfolded protein and plotted as the unfolded fraction *versus* temperature.

### Binding affinity of ICP8Δ60 to CTD fragments

For binding titrations, ICP8Δ60 protein was labeled using the amine reactive Monolith Protein Labeling Kit RED-NHS (NanoTemper Technologies, Catalog #: MO-L001), according to the protocol provided with the kit. Following the labeling reaction, protein concentration was determined using Bradford assays. Samples were prepared by adding increasing concentrations of the purified ICP8-CTD fragment (from low nanomolar to high micromolar) to an equal volume of the labeled ICP8Δ60 protein (20 nM or 50 nM final concentration) in the binding buffer (20 mM sodium phosphate, pH [7.5], 100 mM NaCl, 0.1 mM EDTA, 2 mM DTT, 5 mM MgCl_2_, and 0.05% [v/v] Tween 20) and incubated for 30 min at RT. Rapid scanning of 16 capillaries loaded with fluorescently labeled target protein at a constant concentration and individual fragment in increasing concentration gradients enabled the determination of equilibrium binding constant (K_D_), using a one site binding model. Assays were completed with sample duplicates, and results are reported as the 68.3% confidence intervals of at least two independent experiments. Data were collected using the MO.Control software (NanoTemper Technologies) and analyzed by MO.Affinity Analysis software (NanoTemper Technologies).

### Inhibition of filament formation

ICP8 WT (1.5 μM) was preincubated for 10 min at RT in the presence of 1.5 μM of WT or mutant versions of His tagged-ICP8-CTD or BSA in storage buffer (20 mM sodium phosphate pH [7.5], 100 mM NaCl, 2 mM DTT, 0.1 mM EDTA, and 10% [v/v] glycerol). After 10 min, MgCl_2_ was added to a final concentration of 5 mM, and samples incubated at 4 °C overnight. Samples were then diluted 1:5 in water and absorbed onto Formvar-carbon-coated 300-mesh copper grids and imaged as described above. Filament length was quantified from three randomly chosen 21.4 um^2^ FOVs (100,00×) using ImageJ software.

### Replication compartment formation in the presence of transiently expressed mutant and WT versions of Flag-ICP8-CTD

Vero cells were grown to ∼80 to 90% confluency in 24-well culture plates on 12 mm glass coverslips. Cells were transfected with 60 ng of pcDNA3-Flag-ICP8-CTD WT or mutant versions (CTD-D1087A or CTD-FNF) along with 450 ng of pUC119 carrier DNA plasmid using Lipofectamine 2000 (Invitrogen) following manufacturer’s protocol. Cells transfected with pcDNA3 EV in combination with a plasmid expressing mCherry were used as a control. Fourteen to 16 h post transfection, cells were infected with KOS WT virus at MOI of 10 PFU/cell. Seven hours post infection, cells grown on the coverslips were processed for immunofluorescence staining using the following primary antibodies: monoclonal mouse anti-Flag (1:200) and polyclonal rabbit anti-UL42 clone 833 (1:400). The following secondary antibodies were used for fluorescent detection: Alexa Flour goat anti-mouse 584 and goat anti-rabbit 488. For the visualization of cellular DNA, Hoechst stain was used. Multiple images per coverslip were taken with BioTek Cytation 5 Cell Imaging Multi-Mode Reader using 20×. Flag/mCherry expressing cells mark the transfected cells and presence of UL42 protein is an indication of viral infection and a marker for viral RC. A population of 50 to 200 transfected/infected cells (as determined by expression of Flag/mCherry and UL42 protein respectively) for each transfected plasmid was evaluated for the presence of viral RC. Ratio of cells containing RC to total number of cells counted for each plasmid was normalized to the ratio of the control sample and presented as percent RC formation according to the formula: Ratio _(Flag)_/Ratio_(mCherry)_ × 100.

### Inhibition of virus production in the presence of transiently expressed Flag-ICP8-CTD protein

Vero cells grown to ∼80 to 90% confluency in a 12-well culture plate were transfected with 100 ng of pSAK-ICP8 in combination with 700 ng of carrier DNA, pUC119 and 200 ng of either EV plasmid as control sample or a plasmid expressing Flag-tagged ICP8-CTD (WT or mutant versions). Transfection was performed using Lipofectamine 2000 (Invitrogen) according to manufacturer’s protocol. Fourteen to 16 h post transfection, cells were infected at MOI of 5 PFU/cell with HD-2 virus, and at 24 h post infection, viral yields in supernatants containing released virus were determined by titration on ICP8-expressing Vero cells (S2). Results were presented as a percentage of viral yield calculated by the following formula: viral yield of CTD-transfected sample divided by viral yield of EV-transfected sample x 100.

## Data availability

All data described are contained within the manuscript or the Supporting information file. Raw data will be made available upon request to the corresponding author.

## Supporting information

This article contains supporting information (51, 55, 56).

## Conflict of interest

S. K. W., D. L. W. and L. R. W. are co-founders of Quercus Molecular Design, LLC, an early stage company focused on infectious disease. The other authors declare that they have no conflicts of interest with the contents of this article.
