# Supplementary material for: Two distinct protein-protein interfaces drive cooperative binding of the herpes simplex virus protein ICP8 to ssDNA, filament formation and annealing essential for viral replication
Source: J Biol Chem. 2025 Jul 18;301(9):110498. doi: 10.1016/j.jbc.2025.110498 (PMC12409425; doi:10.1016/j.jbc.2025.110498)
Supplement: Supplementary Material [file mmc1.docx]

**Two distinct protein-protein interfaces drive ICP8 cooperative binding to ssDNA, filament formation and annealing essential for viral replication**

Katherine A. Discipio^†^, Jolanta Krucinska^‡^, Renata Szczepaniak^†^, Heidi Erlandsen^‡^, Andrea M. Makkay^†^, Lee R. Wright^‡^, Dennis L. Wright^‡^ and Sandra K. Weller^†^

**Supplementary Information**

**Supplementary Figure S1.** Thermal stability of ICP8-CTD constructs measured by label-free differential scanning fluorimetry. The first derivative of the fluorescence ratio (∆F350nm/∆F330nm) as a function of temperature is shown. Colored dots represent the maximum of the peak, which is the temperature of inflection (Ti). An average of at least three experiments were performed independently, each done in duplicate. One representative curve for each sample is shown:ICP8-CTD WT (purple), ICP8-CTD D1087A (red), ICP8-CTD FNF→AAA (orange) and ICP8-CTD∆60 (green). The Ti values with standard deviations are shown in Table 1.

**Supplementary Figures S2A and S2B.** Thermal stability of ICP8∆60 upon binding to ICP8-CTD fragments, at fixed 1:10 molar ratio, in the presence and absence of ssDNA (at 1.2 molar excess to ICP8∆60) in 20 mM HEPES (pH 7.6), 100 mM NaCl, 100 µM EDTA, 1 mM TCEP, 10% glycerol, +/- 5 mM MgCl_2_. The first derivative of the fluorescence ratio (∆F350nm/∆F330nm) as a function of temperature is shown in the diagrams. Colored dots represent the points of inflection (Ti) in the presence and absence of different binding partner(s).

**Figure S2A**. The first-derivative curves of ICP8∆60 alone, w/o MgCl_2_ in the binding buffer (green), ICP8∆60 alone with 5 mM MgCl_2_ in the binding buffer (red), ICP8∆60:Mg:ICP8-CTD WT (yellow), ICP8∆60:Mg:ICP8-CTD WT:DNA (blue), ICP8∆60:Mg:ICP8-CTD D1087A (purple) and ICP8∆60:Mg:ICP8-CTD D1087A:DNA (black) at 1:10 molar ratio of ICP8∆60 to the ICP8-CTD fragment.

**Figure S2B**. The first-derivative curves of ICP8∆60 alone, w/o MgCl_2_ in the binding buffer (green), ICP8∆60 alone with 5 mM MgCl_2_ in the binding buffer (red), ICP8∆60:Mg:ICP8-CTD FNF (yellow), ICP8∆60:Mg:ICP8-CTD FNF:DNA (blue), ICP8∆60:Mg:ICP8-CTD delta60 (purple) and ICP8∆60:Mg:ICP8-CTD delta60 :ssDNA (black) at 1:10 molar ratio of ICP8∆60 to the ICP8-CTD fragment.

**Supplementary Figures S3A-D.** Thermal stability of the purified recombinant ICP8∆60 upon binding to the respective ICP8-CTD fragment, prepared at different molar ratios of ICP8∆60 to ICP8-CTD construct. The first derivative of the fluorescence ratio (∆F350nm/∆F330nm) as a function of temperature is shown in the diagrams. Colored dots represent points of inflection (Ti) in the presence and absence of increasing concentration of the binding partner. An average of at least two experiments were performed independently, each done in duplicates and one representative plot for each sample is displayed.

**Figure S3A**. Thermal unfolding profile of the purified recombinant ICP8∆60 dosed with an increasing concentration of the wild-type ICP8-CTD fragment. Thermal shift first-derivative curves of ICP8∆60 alone (black), ICP8-CTD WT alone (red), ICP8∆60:ICP8-CTD WT at 1:2 ratio (orange), ICP8∆60:ICP8-CTD WT at 1:5 ratio (green), ICP8∆60:ICP8-CTD WT at 1:10 ratio (blue) and ICP8∆60:ICP8-CTD WT at 1:20 ratio (purple), analyzed by Tycho software.

**Figure S3B** Thermal unfolding profile of ICP8∆60 dosed with an increasing concentration of the ICP8-CTD D1087A fragment. Thermal shift first-derivative curves of ICP8∆60 alone (black), ICP8-D1087A alone (red), ICP8∆60:ICP8-CTD D1087A at 1:2 ratio (orange), ICP8∆60:ICP8-CTD D1087A at 1:5 ratio (green), ICP8∆60:ICP8-CTD D1087A at 1:10 ratio (blue) and ICP8∆60:ICP8-CTD D1087A at 1:20 ratio (purple), analyzed by Tycho software.

**Figure S3C.** Thermal unfolding profile of ICP8∆60 dosed with an increasing concentration of the ICP8-CTD FNF→AAA mutant. Thermal shift first-derivative curves of ICP8∆60 alone (black), ICP8-CTD FNF→AAA alone (red), ICP8∆60:ICP8-CTD FNF→AAA at 1:2 ratio (orange), ICP8∆60:ICP8-CTD FNF→AAA at 1:5 ratio (green), ICP8∆60:ICP8-CTD FNF→AAA at 1:10 ratio (blue) ) and ICP8∆60:ICP8-CTD FNF→AAA at 1:20 ratio (purple), analyzed by Tycho software .

**Figure S3D.** Thermal unfolding profile of ICP8∆60 dosed with an increasing concentration of the ICP8-CTD ∆60 fragment. Thermal shift first-derivative curves of ICP8∆60 alone (black), ICP8-CTD ∆60 alone (red),ICP8∆60:ICP8-CTD ∆60 at 1:2 ratio (orange), ICP8∆60:ICP8-CTD ∆60 at 1:5 ratio (green), ICP8∆60:ICP8-CTD ∆60 at 1:10 ratio (blue) and ICP8∆60:ICP8-CTD ∆60 at 1:20 ratio (purple), analyzed by Tycho software.

**Supplementary Figures S4A-D.** Thermophoretic analysis of ICP8∆60 interactions with ICP8-CTD constructs. Titration of the non-fluorescent CTD fragment into an equal volume of the RED-NHS labeled ICP8∆60 results in a gradual change in thermophoresis, which is plotted as ∆F*norm* versus ligand concentration to yield a binding curve, that is fitted to obtain the binding constants, K_D_ . The purple zone indicates the cold region (equilibrium phase) and the red zone indicates the hot region (IR laser activation phase). Each circle represents the average of ∆*F_n_*, and error bars show the standard deviation from two individually prepared sample series. The solid line represents the best fit of the data to the 1:1 binding model.

Kd values with 68.3% confidence intervals (CI) are found in Table 3.

1.


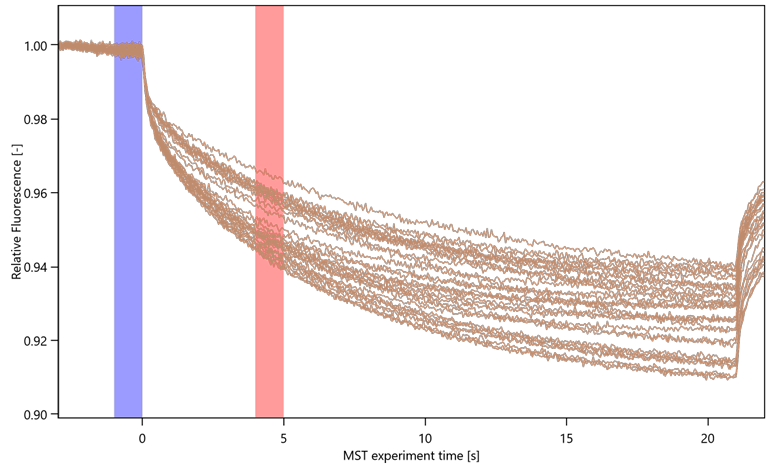


2.


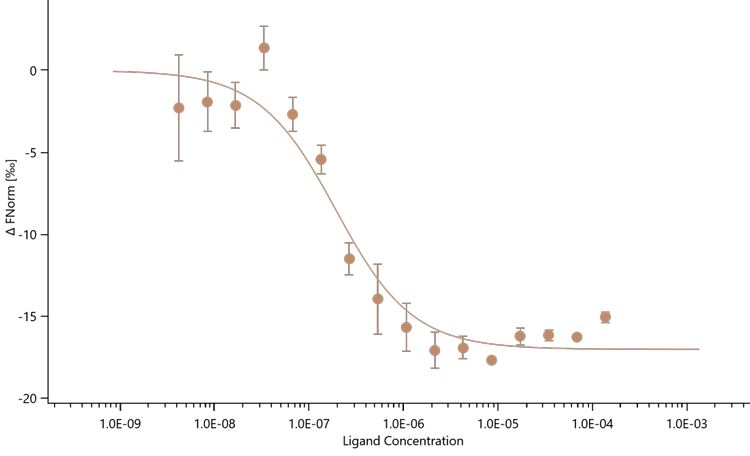


**Figure S4A1-2.** (1) MST traces recorded from capillaries loaded with a dilution series of ICP8-CTD WT, ranging from 138 µM to 0.0042 µM and mixed with 50 nM of the labeled RED-NHS ICP8Δ60. (2) Dose-response binding curve obtained by plotting the ∆F*norm* of merged data of experimental sets of 16 capillaries as a function of ligand concentration using MO. Affinity Analysis software (v3.0.5). The interactions between the wild-type ICP8-CTD construct and the ICP8∆60 induces a pronounced MST signal change, yielding *K*_D_ of 147 nM.

1.


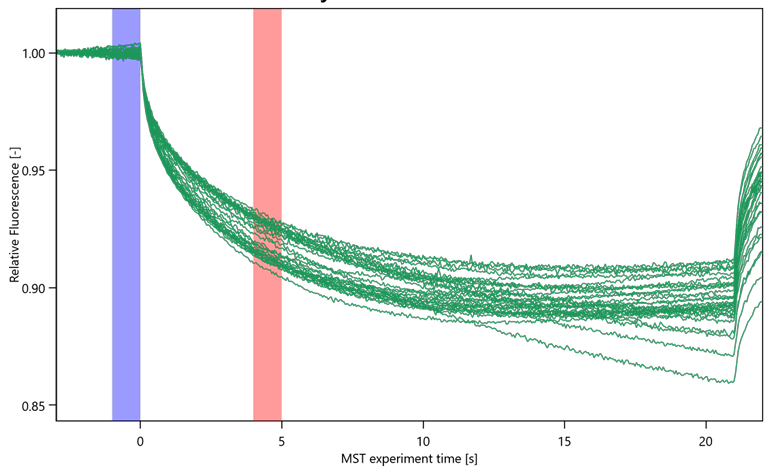


2.


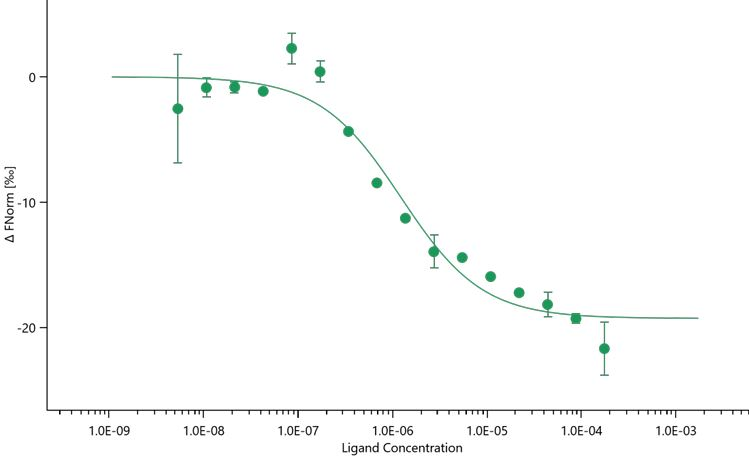


**Figure S4B1-2.** (1) MST traces recorded from capillaries loaded with a dilution series of ICP8-CTD D1087A mutant, ranging from 175 µM to 0.0053 µM and mixed with 20 nM of the labeled RED-NHS ICP8Δ60. (2) Dose-response binding curve obtained by plotting the ∆F*norm* of merged data of experimental sets of 16 capillaries as a function of ligand concentration using MO. Affinity Analysis software (v3.0.5). The interactions between the ICP8-CTD D1087A mutant and the ICP8∆60 produced binding curve with K_D_ value of 1.6 µM.

1.


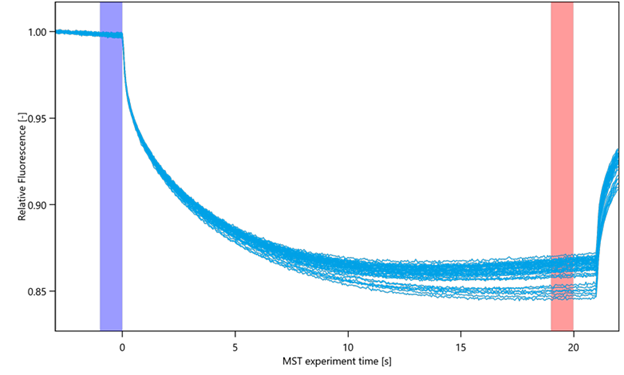


2.


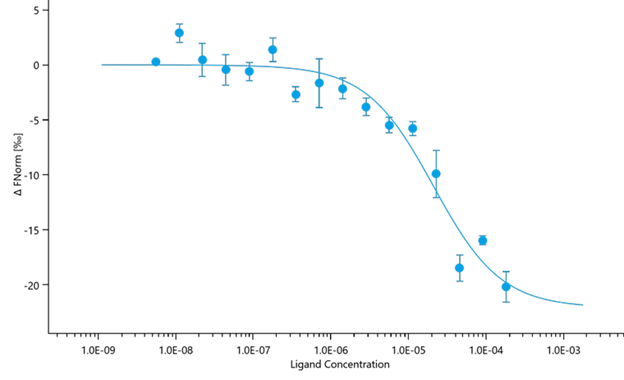


**Figure S4C1-2.** (1) MST traces recorded from capillaries loaded with a dilution series of ICP8-CTD FNF →AAA mutant, ranging from 182 µM to 0.0055 µM and mixed with 20 nM of the labeled RED-NHS ICP8Δ60. (2) Dose-response binding curve obtained by plotting the ∆F*norm* of merged data of experimental sets of 16 capillaries as a function of ligand concentration using MO. Affinity Analysis software (v2.3). The interactions between ICP8∆60 and the ICP8-CTD FNF →AAA mutant gave K_D_ of 21 µM.

1.


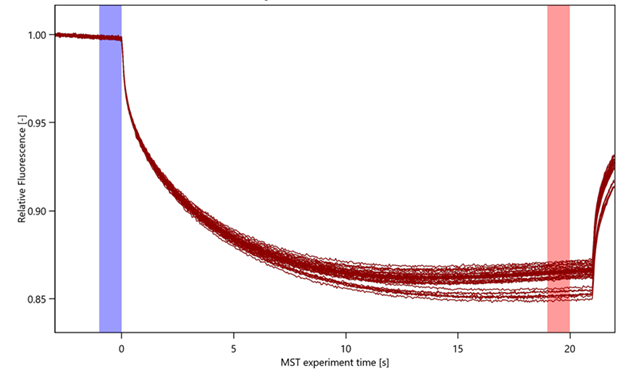


2.


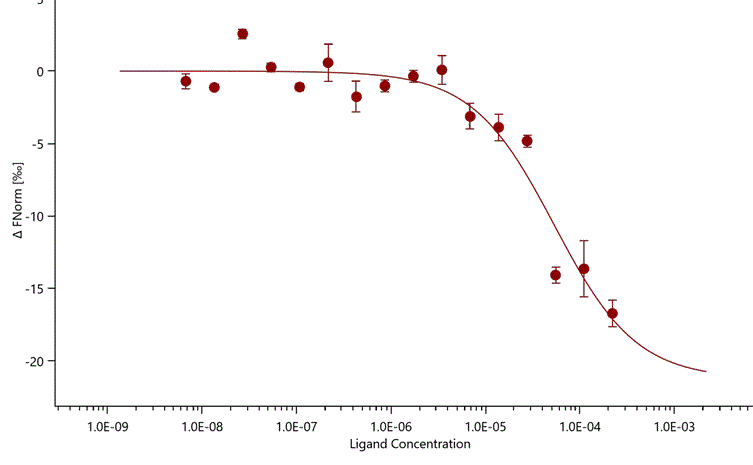


**Figure S4D1-2.** (1) MST traces recorded from capillaries loaded with a dilution series of ICP8-CTD Δ60 mutant, ranging from 221 µM to 0.0067 µM and mixed with 20 nM of the labeled RED-NHS ICP8Δ60. (2) Dose-response binding curve obtained by plotting the ∆F*norm* of merged data of experimental sets of 16 capillaries as a function of ligand concentration using MO. Affinity Analysis software (v2.3). Very weak binding was detected upon titration of ICP8-CTD ∆60 construct into labeled ICP8∆60. The K_D_ equals 54 µM.


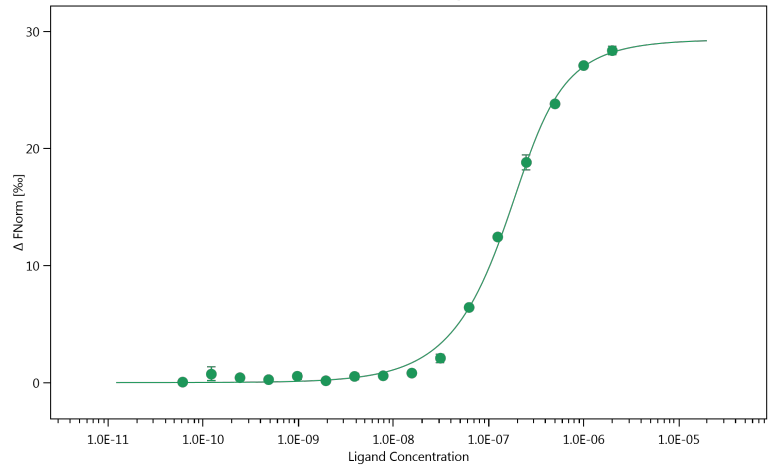


**Figure S5.** MST of the interaction of ICP8∆60 with Cy5-labeled 25-mer poly(dT) DNA. Increasing concentrations of purified protein ICP8∆60, ranging from 2 µM to 0.061 nM were incubated with equal volume of 5´Cy5-labeled 25-mer poly(dT) single stranded DNA (20 nM final concentration) resulting in a strong thermophoretic shift. The calculated K_D_ for this interaction was 70 nM
